# Antibiotic-resistant pathogenic bacterial isolates from patients attending the outpatient department of university of Cape Coast hospital, Ghana: A retrospective study between 2013–2015

**DOI:** 10.1371/journal.pgph.0000417

**Published:** 2022-05-16

**Authors:** Kwame Kumi Asare, Samuel Amoah, Cornelius Agyeman Coomson, Cecil Banson, Derrick Yaro, Jennifer Mbata, Rudolf Aaron Arthur, Peter Bilatam Mayeem, Justice Afrifa, Felicity Bentsi-Enchill, Yeboah Kwaku Opoku

**Affiliations:** 1 Dept. of Biomedical Science, School of Allied Health Sciences, College of Allied Health Sciences, University of Cape Coast, Cape Coast, Ghana; 2 Laboratory Unit, University of Cape Coast Hospital, Cape Coast, Ghana; 3 Dept. of Biology Education, Faculty of Science Education, University of Education, Winneba, Ghana; 4 Offinso College of Education, Ashanti Region, Ghana; 5 Department of Medical Laboratory Science, University of Cape Coast, Cape Coast, Ghana; University of Health and Allied Sciences, GHANA

## Abstract

Uropathogenic *Escherichia coli* (*E*. *coli*) is an important urinary tract infection (UTI) that has been associated with both complicated and uncomplicated disease conditions. The global emergence of multiple drug-resistant (MDR) and extended-spectrum β-lactamase (ESBL) is of public health concern as the resistance limits the current treatment options. The objective of this study was to analyze the antibiotic-resistant patterns among the uropathogenic *E*. *coli* isolates at the University of Cape Coast (UCC) hospital between 2013 and 2015 as baseline data to understand the current antibiotic resistance situation within UCC and its environs. A retrospective cross-sectional study of bacteria isolates at UCC hospital from January 2013 to December 2015 were analyzed. A standard biochemical and antibiotic susceptibility tests were performed using Kirby-Bauer NCCLs modified disc diffusion technique. The network of interaction between pathogenic isolates and antibiotic resistance was performed using Cytoscape software. Statistical significance was tested using ANOVA and one-sample Wilcoxon test. The overall *E*. *coli* prevalence was 15.76% (32/203); females had the highest infection of 17.33% (26/150) compared to male subjects who had 11.32% (6/53) out of all the pathogenic infections. The *E*. *coli* prevalence among the age categories were 2/21 (9.52%), 27/154 (17.53%) and 4/21 (19.05%) among ≤20 years, 21–40 years and 41–60 years respectively. The isolated resistant pathogens exhibited different antibiotic resistance patterns. An interaction network of nodes connecting to other nodes indicating positive correlations between the pathogens and antibiotic resistance was established. *Escherichia coli*, *Citrobacter spp*, *Klebsiella spp* among other isolated pathogens formed higher centrality in the network of interaction with antibiotic resistance. The individual *E*. *coli* isolates showed a significant difference in the mean ± SD (95% CI) pattern of antibiotic resistance, 2.409±1.205 (1.828–2.990), χ2 = 36.68, p<0.0001. In conclusion, the study reports the interaction of *E*. *coli* isolates at UCC hospital and its antibiotic-resistant status between 2013 and 2015. This data forms the baseline information for assessing the current antibiotic status in UCC and its environs.

## Introduction

Antimicrobial resistance is a threat to health. The increasing reports of bacteria resistance to antibiotics limit the treatment options and render commonly encountered bacterial infections such as urinary tract infections (UTIs) difficult to treat. UTIs are invasive or progressive bacterial infections that have exceeded their pathogenic threshold of 105 colony-forming unit (CFU)/mL [1, 2]. UTIs are the third most common bacterial disease associated with the frequent cause of morbidity in both outpatients and hospitalization besides respiratory and gastrointestinal infection [3, 4]. *Escherichia coli*, *Citrobacter species*, *Staphylococcus aureus*, *Staphylococcus faecalis*, *Proteus mirabilis*, *Klebsiella species*, *Streptococcus species* and others are the most frequent cause of UTIs in both developing and developed countries [5, 6]. The adherence of uropathogens to the epithelial is followed by the production of bacteria toxins, which further stimulates an inflammatory response resulting in cell destruction [7, 8]. The cardinal symptoms of bacteria colonization of the urinary tract infections range from cystitis, urethritis, pyelonephritis, hematuria, dysuria, cloudy urine and nocturnal enuresis [9, 10]. Thus, compromising the host immune defence mechanisms and inducing pathogenicity and virulence by pathogenic bacteria [11].

The uropathogenic *E*. *coli* (UPEC) is the most frequently isolated pathogen involved in 25–50% of complicated and more than 70% of uncomplicated UTI cases [12, 13]. The UPEC consist of only a selected number of *E*. *coli* strains that successfully survive, colonize and cause infection in the urinary tract [14]. The UPEC produces virulence factors (VFs) and acquires antibiotic resistance genes required for biofilm formation and nutrient or iron acquisition, invasion of host-cell cytotoxicity or host immune defence and resistance to antibiotic treatment [15–17]. These *E*. *coli* virulence factors are essential for the bacteria to survive in varying environmental conditions, metabolize and use several carbon sources for facultatively anaerobic metabolism [18]. About 20% of female vaginal microbiota constitute UPEC and it is associated with aerobic vaginitis [19, 20].

Improper stewardship, the increased use of unprescribed antibiotics, improper prescription of broad-spectrum antibiotics and their overuse are among the contributing factors for the rapid emergence of antibiotic resistance. The prevalences of *E*. *coli* resistance in high-income countries are Trimethoprim (53.4%), co-amoxiclav (23.6%), ampicillin (8.2%), ciprofloxacin (2.1%) while that of low and middle-income countries are ampicillin (79.8%), co-amoxiclav (60.3%), and ciprofloxacin (26.8%). Antibiotic resistance surveillance in low and middle-income countries lacks. *E*. *coli* resistance to co-trimoxazole (77%), ampicillin (93%) and gentamicin (29%) in several reported cases. In Ghana, *E*. *coli* antibiotic resistance to Amoxicillin, Ampicillin, Cotrimoxazole, Tetracycline, Chloramphenicol, Cefuroxime, Gentamicin, Amikacin, Cefotaxime, and Ciprofloxacin are 100%, 86.8%, 81.6%, 73.6%, 34.42%, 22.6%, 12.74%, 6.97% and 5.7% respectively.

The emergence of antibiotic resistance among the UPEC isolates is a challenge as it substantially influences treatment options [21, 22]. The global emergence and increase of multidrug-resistant (MDR) *E*. *coli* are of prime importance for the management of UPEC [23]. The ESBLs produced by UPEC is able to hydrolyze broad-spectrum cephalosporins and other monobactams [24, 25]. The UPEC antibiotic resistance to cotrimoxazole, ampicillin, nitrofurantoin and fluoroquinolones such as ciprofloxacin and levofloxacin is of major concern [26, 27]. The wide range of UPEC resistance to all classes of antibiotics limits the alternative treatment options [28].

The UPEC antibiotic resistance is not only a threat to nosocomial and healthcare-associated infections but also community infections [29, 30]. The trend and risk factors associated with antibiotic-resistant pathogens associated with UTIs require constant review among bacteria isolates [31, 32]. Such knowledge is essential for policies implementation and the proper usage of antibiotic drugs to combat MDR in UTIs. The study analyzed the antibiotic resistance patterns among pathogenic bacterial isolates from the outpatient department (OPD) at the University of Cape Coast (UCC) hospital between 2013 and 2015 to serve as baseline data to understand the current antibiotic resistance situation within UCC and its immediate environs. The isolated pathogens and their antibiotic-resistant status were visualized using the network of interactions to understand the magnitude and the trends of antibiotic resistance among *species* of pathogenic bacterial isolates.

## Materials & methods

### Study area

A retrospective cross-sectional study of clinical bacteria culture and isolates carried out using the Microbiology Laboratory register records at the University of Cape Coast hospital from January 2013 to December 2015 were analyzed. The university lies between 50 8’ 10” N, 10 17’ 56” W to NE and 50 5’ 51” N, 10 16’ 43”W to SE. The hospital caters for the health needs of students and staff of the university as well as individuals in the surrounding communities. The hospital is situated at the main entrance to the university campus, which is about 160 meters from the shores of the Gulf of Guinea.

### Ethical approval

Ethical approval from the Department of Biomedical Sciences Research Board, University of Cape Coast (DBRB/17/0432) was obtained before the study and permission from the Medical Administrative Committee of the University of Cape Coast Hospital. All data obtained from Laboratory records were anonymous.

### Clinical sample collection and identification of pathogens

Convenience sampling was used for the selection of samples. The study samples included high vaginal swabs, blood samples, urethral swabs, urine samples, stool samples, cerebrospinal fluids samples, ear swabs and wound samples collected at the University of Cape Coast hospital. The patients’ demographic characteristics such as age and sex were also recorded from the registrar. Blood agar (Sigma-Aldrich) and MacConkey agar (Sigma-Aldrich) were used for the isolation of the pathogens. The incubation conditions were 37oC aerobically overnight. The isolates were identified based on colony appearance, Gram stain, catalase-positive, and oxidase negative isolates were further examined.

The bacteria isolates analyzed in this study were obtained using the standard traditional biochemical tests and selective media such as Endo agar (Sigma-Aldrich), MacConkey broth, Simmons citrate agar (Sigma-Aldrich), catalase (Sigma-Aldrich), coagulase (Sigma-Aldrich), oxidase (Sigma-Aldrich), sugar fermentation [Triple sugar iron (TSI) agar] (Sigma-Aldrich), indole (Sigma-Aldrich), citrate utilization (Sigma-Aldrich), urease production (Sigma-Aldrich), and motility tests were used [33]. The *Neisseria gonorrhoeae* confirmatory test was performed using Identicult-Neisseria (Adams Scientific, USA) test [34]. The test depends on beta-galactosidase, gamma-glutamylaminopeptidase and prolyl-hydroxypropyl aminopeptidase and rapid carbohydrate utilization test (RCUT) of clinical isolates of oxidase-positive Gram-negative diplococci. *E*. *coli* confirmatory test was performed using Sorbitol-MacConkey (SMAC) agar with *E*. *coli* O157 antiserum or latex reagents (O157 antibody-coated latex and control latex) based on the manufacturer-recommended procedures.

### Candida *species* detection

Candida *spp* analyzed in this study was obtained by cultivating suspected specimens on Sabouraud glucose agar (SGA) and subsequently confirmed morphologically using assimilation/fermentation methods or cultured Corn meal-Tween 80 agar (CTA)

### Antibiotic Susceptibility Test (AST)

Antimicrobial susceptibility was done by Kirby–Bauer disc diffusion method on Mueller–Hinton agar. Briefly, the bacteria turbidity was measured by comparing the normal saline emulsified pure colonies and 0.5 McFarland solution. The susceptibility of bacteria to Ampicillin (Amp) (10μg), Gentamicin (GEN) (10μg), Cotrimoxazole (COT) (25μg), Cefuroxime (CRX) (30μg), Erythromycin (ERY) (5μg) and Amikacin (AMK) (30μg), amoxicillin/clavulanic acid (AMC: 30 μg), ceftazidime (CAZ) (30 μg), imipenem (IPM) (10 μg), Cefixime (CXM) (30μg), Cefotaxime (CTX) (30μg), Penicillin (PEN) (10 IU), Cloxacillin (CXC) (5μg), ertapenem (ETP) (10 μg), meropenem (MEM) (10 μg), tetracycline (TET) (30 μg), trimethoprim-sulfamethoxazole (SXT) (30μg), gentamicin (GEN) (10μg), nalidixic acid (NAL) (30 μg), ciprofloxacin (CIP) (5 μg), chloramphenicol (CHL) (30 μg), aztreonam (AZT) (15 μg), piperacillin/tazobactam (TZP) (100/10 μg), fosfomycin (FOF) (200 μg), and colistin (CST) (10 μg) were examined using Mueller-Hinton agar antibiotic diffusion technique (Kirby-Bauer NCCLS modified disc diffusion technique). CTX, AMC and CAZ antibiotic discs for double-disk synergy test of ESBLs were also performed. The isolated bacteria were plated on a dry Mueller Hinton agar plate with appropriate antimicrobial-impregnated disks and cultured overnight at 35°C. The antibiotic inhibition zones were measured from the centre to the distinct edges of antibiotic inhibition zones using a ruler with *E*. *coli* ATCC 25922 strain as a control. The AST discs were obtained from Becton Dickinson (BD, Sparks, MD, USA).

### Diagrammatic presentation of relationships between pathogenic bacterial isolates and antibiotic resistance

The network tool (Cytoscape) reduces the underlying complexity in pathogens and antibiotic resistance association through the diagrammatic representation for concepts and relationships. The analysis assessed the characteristics of the isolated pathogens and antibiotic resistance patterns through metrics measurements such as degree, clustering coefficient, shortest paths, centrality, density. The network analysis provides visualizing of the direct association between pathogens and their antibiotic resistance status. It also estimates pathogens and their trends of antibiotic resistance.

### Statistical analysis

The data was entered, validated and analyzed using Excel 2016 (Microsoft Corporation) and GraphPad Prism 9.0.2 software. The network of the interaction of isolates and antibiotic resistance were performed using Cytoscape version 3.8.2 software. The statistical significance was tested using ANOVA, and a one-sample Wilcoxon test at p = 0.05.

## Result

### Demographic characteristics of the patients

A total of 1515 samples were cultured between the years 2013–2015 at the UCC hospital Laboratory with a slightly predominant female prevalence of 51.33%, 69.20% and 63.36% for 2013, 2014 and 2015 respectively. The median (95% CI) for the age 21–40 years for 2013 was 26 (25–28), 2014 was 26 (25–27) and 2015 was 25 (25–26). High vaginal swab (40.64%, 39.45% & 36.64%) and urine (37.97%, 58.85% & 55.22%) were the most frequent sample collected from 2013–2015 (Table 1). The trends of pathogens isolation were high among females compared to males and the median age between subjects ranged between 20 and 35 years (S1 Table).

**Table 1:** Demographic characteristics of the patients.

	2013	2014	2015
**Sex n/N (%)**			
Male	91/187 (48.66)	235/763 (30.80)	207/565 (36.64)
Female	96/187 (51.33)	528/763 (69.20)	358/565 (63.36)
**Age n/median (95% CI)**			
≤20 years	24/18 (15–20)	131/18 (16–19)	113/17 (12–19)
21–40 years	113/26 (25–28)	513/26 (25–27)	344/25 (25–26)
41–60 years	40/49 (45–50)	88/50 (48–51)	68/49 (48–51)
61–80 years	10/71 (61–75)	31/70 (66–73)	40/69 (67–73)
**Isolates n (%)**			
Pathogenic bacteria	93 (49.73)	223 (29.23)	147 (26.02)
Commensal bacteria	30 (16.04)	113 (14.81)	50 (8.85)
Fungi infection	19 (10.16)	97 (12.71)	72 (12.74)
**Specimen n (%)**			
HVS	76 (40.64)	301 (39.45)	207 (36.64)
Urethral	40 (21.39)	13 (1.70)	46 (8.14)
Urine	71 (37.97)	449 (58.85)	312 (55.22)

### An increase in multiple antibiotic-resistant *Escherichia coli* infections among female patients between the ages 21–40 years

**Fig 1:**
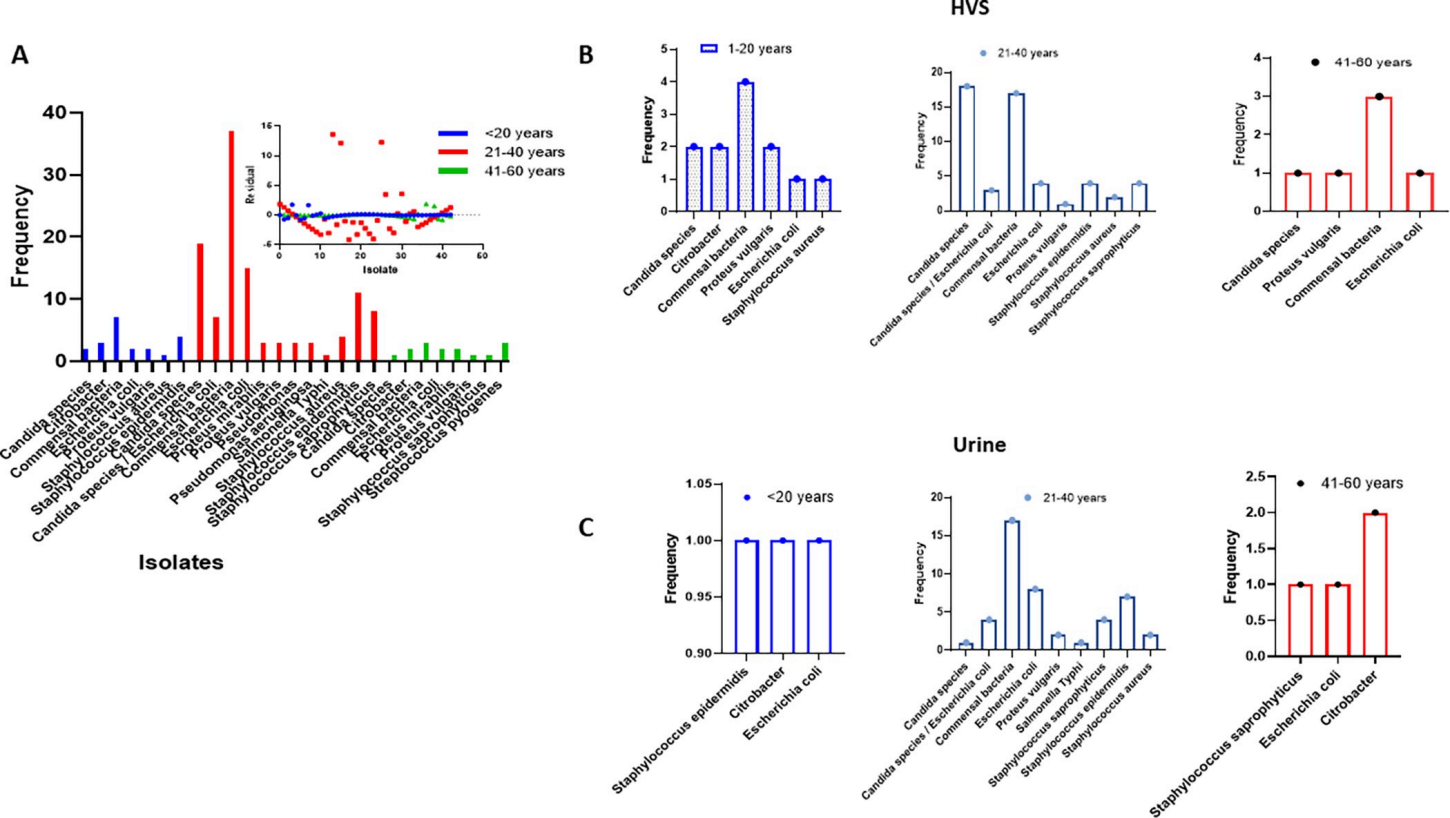
An increased urogenital antibiotic-resistant *Escherichia coli* infection among females between the ages 21–40 years old. A. The trends in the bacteria isolate among female patients with age categories ≤ 20 years, 21–40 years and 41–60 years. The residual plot analysis showed changes in the trends of bacteria isolates among the age 21–40 years. B. The frequency of bacteria isolates from different specimen types across the age categories ≤ 20 years, 21–40 years and 41–60 years. C. The interleaved bars showing frequencies of bacteria isolates from high vagina swabs (HVS) samples across the age ≤ 20 years, 21–40 years and 41–60 years.

Among the pathogenic isolates, the overall prevalence of *E*. *coli* was 17.33% (26/150) and 11.32% (6/53) for females and males respectively. Among the female patients, the age group 21–40 years recorded the highest burden of the number of isolated pathogens compared to ≤20 years and 41–60 years age groups. The age group 21–40 years had 12 (44.44%) different pathogenic isolates compared to 7 (25.93%) and 8 (29.63%) isolates for the age groups ≤20 and 41–60 years. The *E*. *coli* isolates were 2/21 (9.52%), 22/114 (19.30%) and 2/15 (13.33%) among ≤20 years, 21–40 years and 41–60 years respectively. The one-sample Wilcoxon test showed significant difference among the *E*. *coli* isolates compared to other pathogenic bacteria W = 28.0, 2.0 (1.0–7.0), p = 0.0156; W = 78.0, median (95% CI), 5.50 (3.0–15.0), p = 0.0005 and W = 36.0, 2.0 (2.0–3.0), p = 0.0078 among ≤20 years, 21–40 years and 41–60 years respectively. The residual plot showed a dynamic variation pattern of bacterial infections across the age of 21–40 years (Fig 1A). The prevalence of *E*. *coli* infection isolates from HVS among the age categories ≤20 years, 21–40 years and 41–60 years were 8.33%, 7.55% and 16.67% respectively. Again, a coinfection of 5.66% was recorded for *E*. *coli* & *Candida spp* for the age group 21–40 years (Fig 1B). The pathogenic infections observed among the urine samples collected from female patients across the age categories ≤20 years, 21–40 years and 41–60 years showed a similar pattern to that of HVS infections. The number of pathogenic isolates was very low among the age categories ≤20 years with *E*. *coli* infection representing 33.33% of the isolates. The prevalence of *E*. *coli* from the urine specimens among the age groups 21–40 years and 41–60 years were 17.39% and 25.0% respectively (Fig 1C). Notably, more pathogenic bacteria isolates were recorded from the HVS (57.25%) and urine (19.85%) specimens compared to the other forms of specimens collected. The one-sample Wilcoxon statistic showed a significant difference in the median (95% CI), 8.0 (7–75.0), W = 21.0, p = 0.0313 among the specimens analysed (Table 1).

### An increase in multiple antibiotic-resistant *Escherichia coli* infections among male patients between the ages 21–40 years

**Fig 2:**
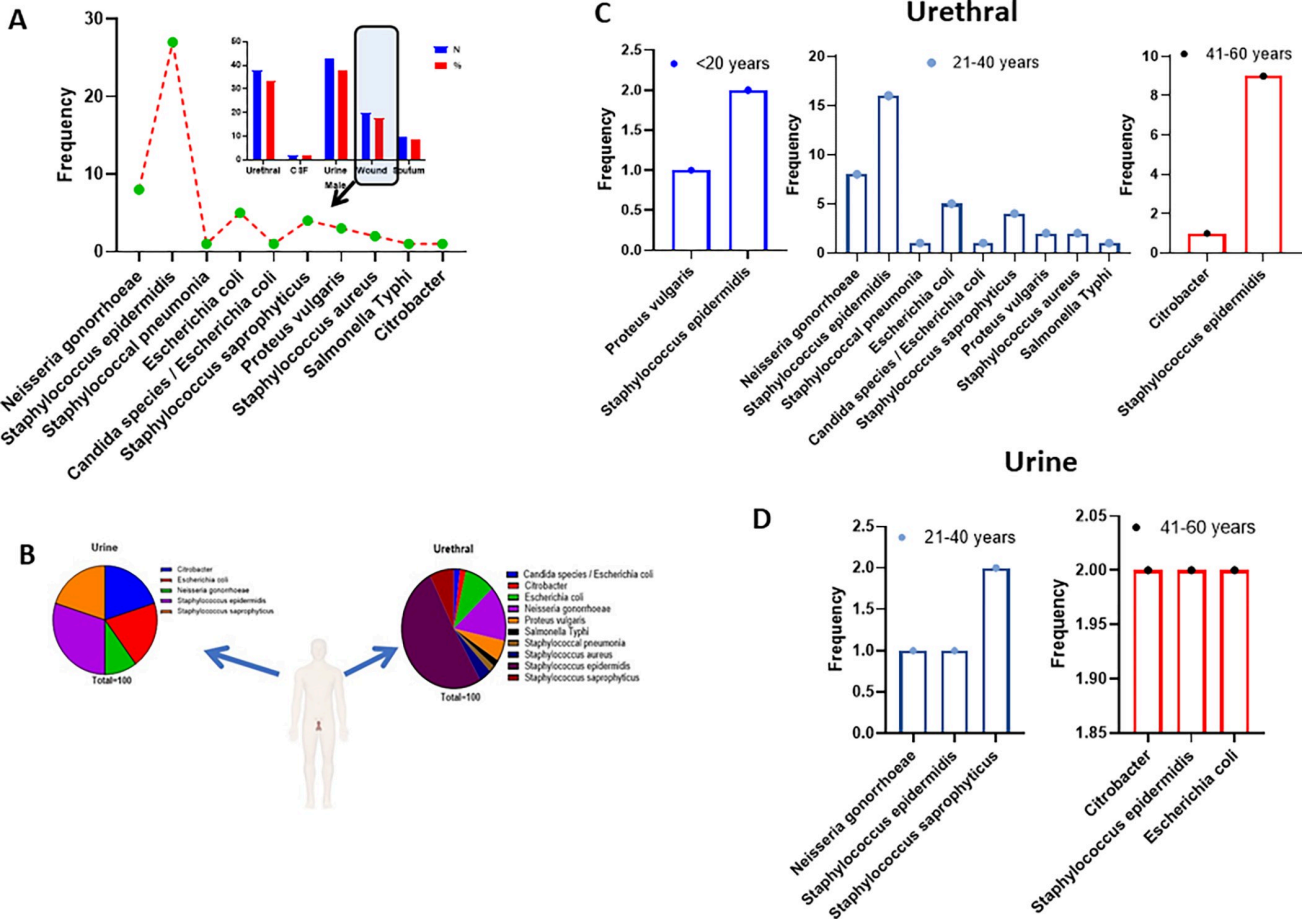
High isolation of multiple antibiotic-resistant *E*. *coli* from urethral swab among male patients between 21–40 years of age. A. The frequency of different bacteria isolates from urethral swab samples. 50.94% of the isolates were *Staphylococcus epidermidis*, 15.09% were *Neisseria gonorrhoeae* and 9.43% *Escherichia coli* being the third-highest bacteria isolates from the male urethra. B. Pie charts showing the various % frequencies of bacteria isolated from the different specimens collected from male patients attending UCC hospital. *Escherichia coli* were only isolated from urethral swabs (9.43%) and urine (20%) among the respective bacteria isolates from each specimen. C. The interleaved bars showing frequencies of bacteria isolates from Urethral swabs samples from male patients across the age categories ≤ 20 years, 21–40 years and 41–60 years. D. The interleaved bars show frequencies of bacteria isolates from urine samples across the age categories 21–40 years and 41–60 years. No bacteria isolates were obtained among the age ≤ 20 years.

Even though the majority of the male samples analysed were from urine (38.05%) followed by urethral swabs (33.63%), the urethral swabs had the largest number of different pathogenic bacteria isolates. *S*. *epidermidis* (50.94%), *N*. *gonorrhoeae* (15.09%), *E*. *coli* (9.43%) and *S*. *saprophyticus* (7.55%) were the most dominant pathogenic bacteria isolated from the male urethral. The one-sample Wilcoxon test showed a significant difference among the isolates, median (2.50), W = 55.0, p = 0.002 (Fig 2A). The different specimens collected from the male patients had different pathogenic bacteria infections with variations in their prevalence. A prevalence of 9.43% and 20.0% *E*. *coli* isolates were recorded from urethral and urine samples respectively (Fig 2B). The prevalence of *E*. *coli* from the urethra among the ages between 21–40 years was 12.5% (5/40) (Fig 2C). *E*. *coli* 33.3% (2/6) was only isolated from the urine samples among the ages 41–60 years in the male subjects (Fig 2D).

### The network of interactions of pathogenic bacteria isolates and antibiotic resistance

**Fig 3:**
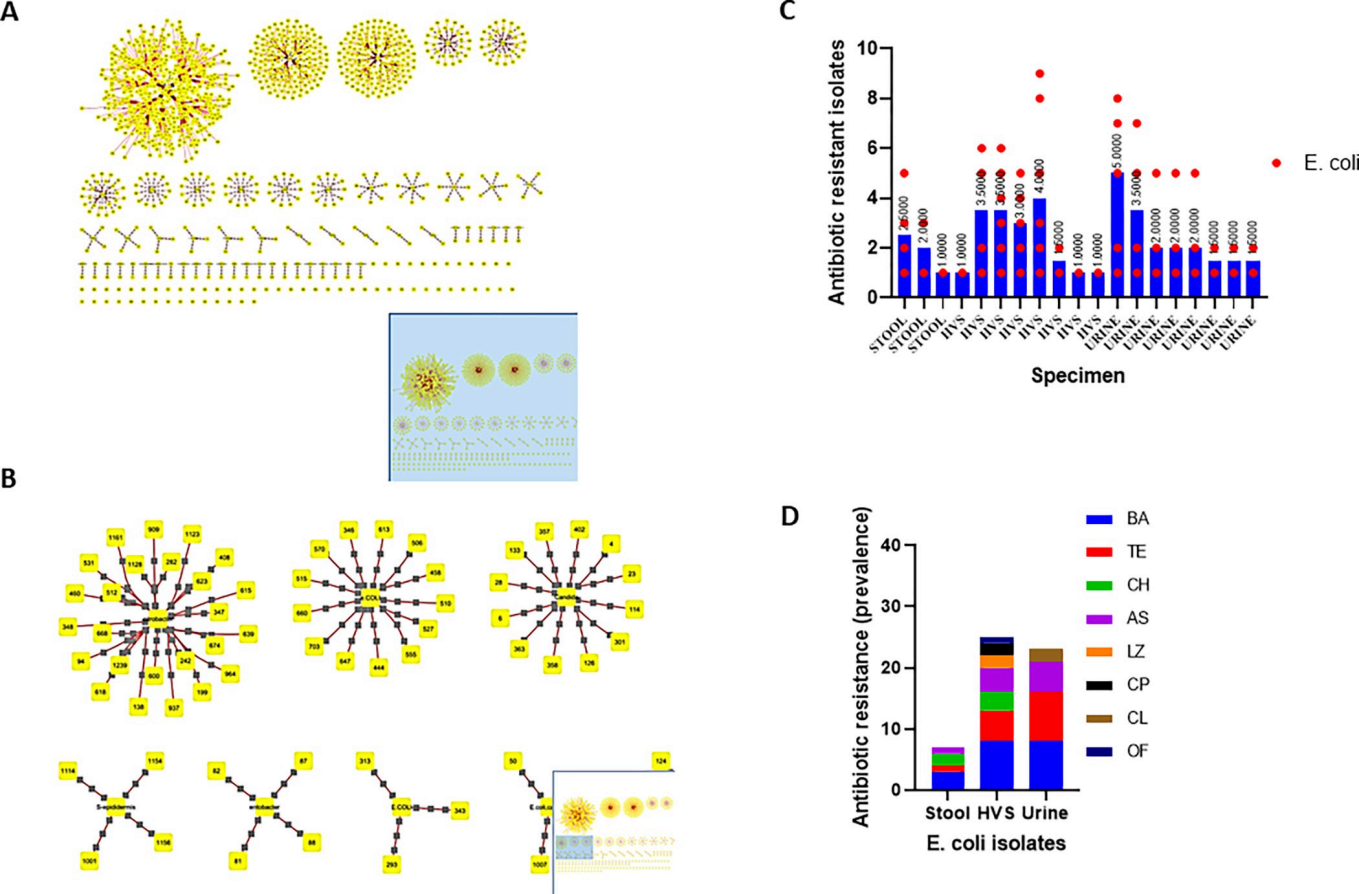
A unique and separate interactions of bacteria isolates and antibiotic resistance pattern. A. Bacteria isolates interaction network, in which nodes represent different associations with antibiotic resistance and bacteria isolates. B. Zoom-in of the interaction networks showing individual bacteria isolates forms a separate and unique interaction. The *Escherichia coli* and *Citrobacter species* showed interesting interaction networks. C. The different antibiotic resistance patterns of *Escherichia coli* isolates from the stool, High vaginal swabs (HVS) and urine specimens. The bar graph is the mean *E*. *coli* isolates with similar antibiotic resistance pattern and each dot represents the mean of the individual antibiotics. D. The stacked bars show the various antibiotics and the prevalence of resistant *Escherichia coli* isolates from stool, HVS and urine. Bactrim (BA), tetracycline (TE), ampicillin-sulbactam (AS), clindamycin (CL), ofloxacin (OF), lincomycin (LZ) and chloramphenicol (CH).

The isolated resistant pathogens exhibited different antibiotic resistance patterns (S2 Table). To assess the interaction between the pathogenic isolates and antibiotic resistance, an interaction network analysis was performed. The result showed a network of nodes connecting to other nodes indicating positive correlations between these pathogens and their sensitivity to various antibiotics (Fig 3A). The individual pathogenic isolates formed a separate cluster of interactions (edges) with the various antibiotics and there was no direct interaction between the individual isolated pathogens. *E*. *coli*, *Citrobacter spp*, *Klebsiella spp* and the other isolated pathogens formed higher centrality in the network of interaction with antibiotic resistance (Fig 3B). This indicates the difference in the levels of antibiotic resistance among the individual pathogens. To understand the interactions, the individual *E*. *coli* isolates from stool, HVS and urine were further analyzed for their antibiotic resistance patterns. The individual *E*. *coli* isolates showed a significant difference in the mean ± SD (95% CI) pattern of antibiotic resistance, 2.409±1.205 (1.828–2.990), χ2 = 36.68, p<0.0001 (Fig 3C). The prevalence of Bactrim (BA), tetracycline (TE), ampicillin-sulbactam (AS), & chloramphenicol (CH) antibiotic resistance were 34.55%, 25.45%, 18.18% & 9.09% among the isolated *E*. *coli*. Furthermore, 42.11% vs 42.11%, 35.71 vs 57.14%, 60% vs 0% and 40% vs 50% were recorded as the antibiotic resistance pattern for BA, TE, CH and AS among the *E*. *coli* isolated from HVS vs urine specimen (Fig 3D). Type III two-way ANOVA analysis showed that *E*. *coli* isolates from stool, HVS and urine exhibit a significant difference in their antibiotic resistance patterns F = 5.399, p = 0.0408.

## Discussion

Multiple drug resistance (MDR) pathogenic bacteria associated with severe morbidity in UTIs is a major global health concern [35, 36]. Antibiotic resistance and rapid spread of aminoglycosides and the lactams such as cephalosporins and fluoroquinolone among uropathogenic bacteria undermines the clinical management of the infection and result in a poor prognosis [37, 38]. The development of resistance to the third-generation cephalosporins and nitrofurantoin are a major cause of prolonging hospitalization among infected patients and limiting bacteria treatment options [39, 40]. The antibiotic-resistant uropathogens employ several resistance mechanisms including the expressions of β-lactamases, TEM-, SHV-, CTX-M-, and OXA-type resistance genes responsible for ESBLs [41, 42]. ESBLs producing bacteria differs from patients, clinical and geographical settings even though the main source of ESBL producing bacteria are nosocomial [43]. This study reports on uropathogenic *E*. *coli* infections among patients visiting the University of Cape Coast hospital from January 2013 to December 2015 and their antibiotic-resistant patterns.

The study showed that *E*. *coli* significantly colonized the female vaginal and male urethral compared to other pathogens and the majority of UPEC colonization were observed among the 21–40 years category. Studies have documented the association of *E*. *coli* colonization of the vagina with very low birth weight and preterm delivery [44, 45]. Also, the vagina and male urethra colonization of the sperm agglutinating strain of *E*. *coli* induces infertility and treatment with antibiotics improves the quality of semen and increases fertility among females [46, 47]. Coincidentally, 21–40 years, which happens to be the fertile stage happens to be the most frequently affected by *E*. *coli* infection.

*E*. *coli* adhere and colonize the uroepithelial cells, resist urinary stream elimination, and avoid specific immune reactions [48, 49]. *E*. *coli* infections cause disease by stimulating cytotoxic activities and local and systemic inflammatory responses [50, 51]. The stimulated local inflammation causes the inflex of granulocytes, macrophages, and monocytes into the tissue [52]. This causes the secretion of proinflammatory cytokines such as IL-1, IL-6, IFN-γ, TNF-ɑ, and infiltration of polymorphonuclear cells to activate an acute-phase response [53].

Uropathogenic *E*. *coli* is reported to cause most of the clinically important UTIs globally with a considerably high resistance rate to amoxicillin, tetracycline, and trimethoprim/sulfamethoxazole [54]. The simultaneous resistance of *E*. *coli* to several antibiotics has become a worldwide problem. The network analysis showed unique interactions between individual pathogenic isolates and their resistance to the various antibiotics. Ciprofloxacin (Cipro), ofloxacin (Floxin), trimethoprim-sulfamethoxazole (TMP-SMX; Bactrim, Septra), and nitrofurantoin (Furadantin) antibiotics have been used for the treatment and eradication of UTIs [55]. However, treatment failures of *E*. *coli* to TMP-SMX had been associated with resistance to these antibiotics [56, 57].

The Surveillance Network (TSN) database has previously reported 38%, 17%, 0.8% and 1.9–2.5% *E*. *coli* resistance respectively to ampicillin, TMP-SMX, nitrofurantoin, and fluoroquinolones [58, 59]. In this study, *E*. *coli* resistance to Bactrim (BA) was the highest followed by tetracycline and ampicillin-sulbactam (AS) or clindamycin (CL). Again, these UTI isolates exhibited multidrug resistance. The fluoroquinolone antibiotics have been used for the treatment of resistant *E*. *coli* in uncomplicated UTIs as a first-line drug in communities that have reported 10–20% resistance to TMP-SMX [60]. However, several studies have reported resistance to ciprofloxacin and ofloxacin [61]. In this study, we also report an observed *E*. *coli* resistance to ciprofloxacin and ofloxacin.

Antibiotic-resistant *E*. *coli* isolated from the urine or the urinary tract is of clinical significance due to a high concentration of antibiotics found in the urine samples [56, 62]. The resistance of TMP-SMX has been steadily increasing in developing countries due to the easy transfer of high-level plasmid encoding the resistance genes for both trimethoprim and sulfamethoxazole among gram-negative urinary tract infections [16, 63]. The TMP-SMX resistance had been predicted to emerge rapidly using mathematical modelling even with very small quantities of resistance genes and plasmids [64]. Similarly, the continuous use of these antibiotics in a population is directly related to the persistence of resistance in an environment [65].

The decrease in the first-line antibiotics efficacy, increased hospitalization, morbidity, and mortality is a global public health concern [66]. There is a limitation in quantifying the extent of antibiotic resistance as there is not enough information on the efficacy of antibiotic drugs for the treatment of *E*. *coli* infections [67]. Currently, the status and the severity of UTIs in the Central region of Ghana remains unknown. Thus, it is important to understand the severity of the UTIs, virulence factors (VFs) in UPEC isolates, and the risk factors that influence antibiotic resistance in the central region of Ghana. This retrospective study serves as the baseline information to assess current antibiotic resistance status in the University of Cape Coast and its environs in the Central region of Ghana.

The limitation of this study is that the results reported in this manuscript do not reflect the current UTIs and antibiotic resistance status as the pathogenic infections pattern, diversity and treatment regimens may have changed with time. However, this information is important to understand the basis for the evolution of ESBL and MDR antibiotic resistance among the UPEC.

In inclusion, this retrospective study reports the antibiotic resistance status of *E*. *coli* isolates from the urinary tract infection. We also report on the pattern of pathogens associated with UTIs, the interaction of the isolates and the various antibiotic resistances among patients visiting the UCC hospital.

## Supporting information

S1 Table The patterns of isolation of resistant pathogens among males and females at the UCC hospital department. (XLS)

S2 Table The antibiotics zone of inhibitions among the resistant pathogens isolated from 2013 to 2015. (XLS)
