# Supplementary material for: Antibiotic-resistant pathogenic bacterial isolates from patients attending the outpatient department of university of Cape Coast hospital, Ghana: A retrospective study between 2013–2015
Source: PLOS Glob Public Health. 2022 May 16;2(5):e0000417. doi: 10.1371/journal.pgph.0000417 (PMC10021532; doi:10.1371/journal.pgph.0000417)
Supplement: Attachment — Submitted filename: 2.12.2021 Response to reviewers_Microbiology.doc [file pgph.0000417.s003.doc]

Response to reviewers’ comments

5. Review Comments to the Author

Please use the space provided to explain your answers to the questions above. You may also include additional comments for the author, including concerns about dual publication, research ethics, or publication ethics. (Please upload your review as an attachment if it exceeds 20,000 characters)

Reviewer #1: This study is very essential as it reports the frequencies and prevalence of bacterial DR isolates in urogenital specimens in the Central region of Ghana, particularly, in hospital attendees in the UCC Hospital. The use of cytoscape in presentations of data was interesting. However, the figures were not clear (especially Fig 2B, 3A and B) and it will be better if clear single figures are presented for all the images.

Response: we have revised the figures and have also included separate Figure 2B, Figure 3A and Figure 3 B

Abstract
1. Introduction: separate spectrum from β-lactamase

Response: We revised it to “extended spectrum β-lactamase (ESBL)”

2. Results: The E. coli prevalence among the age categories were 2/21 (9.52%), 22/114 (19.30%) and 2/15 (13.33%) among ≤20 years, 21-40 years and 41-60 years respectively, the numerators don’t add up to the 32 infections. It looks as if you presented for only the females as the numerator add up to 26 and the denominator adds up to 150. Please recheck and revise

Response: we have revised the statement to “The *E. coli* prevalence among the age categories were 2/21 (9.52%), 27/154 (17.53%) and 4/21 (19.05%) among ≤20 years, 21-40 years and 41-60 years respectively.”

Introduction
1. … their pathogenic threshold of 105 CFU/mL. Indicate the full form of abbreviation on initial use

Response: CFU/mL has been revised to “colony-forming unit (CFU)/mL”

2. UTIs are the third most common bacterial disease, indicate the first two most common bacterial diseases. This will enrich the background of the study

Response: We have revised to include the first two most common bacterial diseases to read “UTIs are the third most common bacterial disease associated with the frequent cause of morbidity in both outpatients and hospitalization beside respiratory and gastrointestinal infection [3, 4]”

3. The mechanism of development of bacterial drug resistance was poor explained. Please explain into details mechanisms by which bacteria may become insensitive to antibiotics. The molecular basis was provided but there are human behaviors that encourage bacterial drug resistance.

Response: We have included these statements “Improper stewardship, the increased use of unprescribed antibiotics, improper prescription of broad-spectrum antibiotics and overuse are among the contributing factors for the rapid emergence of antibiotic resistance.”

4. Additionally, review global, continental and local trend of DR and some economic importance of DR regarding bacterial infections

Response: we have included the statement “The prevalences of E. coli resistance in high-income countries are Trimethoprim (53.4%), co-amoxiclav (23.6%), ampicillin (8.2%), ciprofloxacin (2.1%) while that of low and middle-income countries are ampicillin (79.8%), co-amoxiclav (60.3%), and ciprofloxacin (26.8%). Antibiotic resistance surveillance in low and middle-income countries lacks. E. coli resistance to co-trimoxazole (77%), ampicillin (93%) and gentamicin (29%) in several reported cases. In Ghana, E. coli antibiotic resistance to Amoxicillin, Ampicillin, Cotrimoxazole, Tetracycline, Chloramphenicol, Cefuroxime, Gentamicin, Amikacin, Cefotaxime, and Ciprofloxacin are 100%, 86.8%, 81.6%, 73.6%, 34.42%, 22.6%, 12.74%, 6.97% and 5.7% respectively.”

5. The trend and risk factors associated with antibiotic resistance pathogens associated with UTIs require constant, change resistance to resistant

Response: Resistance has been changed to “resistant”

Methods
1. Study clinical sample collection …, authors should indicate the sampling strategy employed in this study whether random or convenient

Response: We have revised to include sampling strategy to read “Convenience sampling was used for the selection of samples.”

2. Under the objective of your study was to look at E. coli infections in urosamples. It is not clear why under Clinical sample collection and identification of pathogens, you indicated that pathogens isolated in blood samples, stool samples, cerebrospinal fluids samples, ear swabs and wound samples were also analyzed.

Response: We have revised to include all the data analyzed. The title have been revised to read “**Antibiotic Resistance among pathogenic bacterial isolates from patients attending the Outpatient Department of University of Cape Coast hospital, Ghana: A retrospective study between 2013-2015**”

2b. Under the same subheading, you indicated that ‘All the samples were spread onto Blood agar (Sigma-Aldrich) and MacConkey agar (Sigma-Aldrich) and incubated at 37°C overnight’. My understanding was that you reviewed laboratory records so its not proper to indicated how the C/S was done in this way. you will have to review that statement to reflect that blood agar (Sigma-Aldrich) and MacConkey agar (Sigma-Aldrich) was used to isolate the pathogen analyzed in this study. They incubation conditions were 37°C aerobically overnight.

Response: the statement has been revised to “Blood agar (Sigma-Aldrich) and MacConkey agar (Sigma-Aldrich) were used for the isolation of the pathogens. The incubation conditions were 37^o^C aerobically overnight.”

3. Under Biochemical Identification, avoid repeating the source of the reagents (Sigma-Aldrich). Revision is required in that regard

Response: We have revised and removed all repeated reagent sources

4. Change (Triple sugar iron (TSI) agar) to [Triple sugar iron (TSI) agar]

Response: (Triple sugar iron (TSI) agar) has been changed to [Triple sugar iron (TSI) agar]

5. MacConkey broth (Sigma-Aldrich), and Simmons citrate agar (Sigma-Aldrich) … no need to insert and between these 2 media while others were to follow

Response: And has been removed to read “MacConkey broth, Simmons citrate agar (Sigma-Aldrich), catalase (Sigma-Aldrich),”

6. As was dine for the other, indicate the source of these reagents; catalase, oxidase, sugar fermentation (Triple sugar iron (TSI) agar), indole, citrate utilization, urease

Response: We have included the reagent sources as shown “selective media such as Endo agar (Sigma-Aldrich), MacConkey broth, Simmons citrate agar (Sigma-Aldrich), catalase (Sigma-Aldrich), coagulase (Sigma-Aldrich), oxidase (Sigma-Aldrich), sugar fermentation [Triple sugar iron (TSI) agar) (Sigma-Aldrich), indole (Sigma-Aldrich), citrate utilization (Sigma-Aldrich), urease production (Sigma-Aldrich), and motility tests were used. The *Neisseria gonorrhoeae* confirmatory test was performed using Identicult-Neisseria (Adams Scientific, USA) test [34].”

7. Under Biochemical Identification, revise the section to reflect that, that was the technique used to obtain the isolates used in this study. You may be working in the hospital lab and may be familiar with the protocols for bacteria isolation and identification, you don’t have to indicate as if you purposively isolated these pathogens, remember, this is a retrospective study (analysis of already isolated and identified pathogens)

Response: We have revised this section and it has been included in the subsection “**Clinical sample collection and identification of pathogens**” as “ The bacteria isolates analyzed in this study were obtained using the standard traditional biochemical tests and selective media such as Endo agar (Sigma-Aldrich), MacConkey broth, Simmons citrate agar (Sigma-Aldrich), catalase (Sigma-Aldrich), coagulase (Sigma-Aldrich), oxidase (Sigma-Aldrich), sugar fermentation [Triple sugar iron (TSI) agar) (Sigma-Aldrich), indole (Sigma-Aldrich), citrate utilization (Sigma-Aldrich), urease production (Sigma-Aldrich), and motility tests were used. The *Neisseria gonorrhoeae* confirmatory test was performed using Identicult-Neisseria (Adams Scientific, USA) test [34]. The test depends on beta-galactosidase, gamma-glutamylaminopeptidase and prolyl-hydroxypropyl aminopeptidase and rapid carbohydrate utilization test (RCUT) of clinical isolates of oxidase-positive Gram-negative diplococci*. E. coli* confirmatory test was performed using Sorbitol-MacConkey (SMAC) agar with *E. coli* O157 antiserum or latex reagents (O157 antibody-coated latex and control latex) based on the manufacturer-recommended procedures.”

8. Candida species detection: you can revise this subsection as … Candida spp analyzed in this study was obtained by cultivating suspected specimens on Sabouraud glucose agar (SGA) and subsequently, the isolates were confirmed morphologically using assimilation/fermentation methods or cultured Corn meal-Tween 80 agar (CTA)

Response: The subsection has been revised to “Candida spp analyzed in this study was obtained by cultivating suspected specimens on Sabouraud glucose agar (SGA) and subsequently, the isolates were confirmed morphologically using assimilation/fermentation methods or cultured Corn meal-Tween 80 agar (CTA)”

9. Under Antibiotic Susceptibility Test (AST), previous comments apply to this section as well

Response: This section has been revised to “Antimicrobial susceptibility was done by Kirby–Bauer disc diffusion method on Mueller–Hinton agar. Briefly, bacteria turbidity was measured by comparing the normal saline emulsified pure colonies to 0.5 McFarland solution. The susceptibility of bacteria [33] to Ampicillin (Amp) (10μg), Gentamicin (GEN) (10μg), Cotrimoxazole (COT) (25μg), Cefuroxime (CRX) (30μg), Erythromycin (ERY) (5μg) and Amikacin (AMK) (30μg), amoxicillin/clavulanic acid (AMC: 30 μg), cefuroxime (CXM: 30 μg), cefotaxime (CTX: 30 μg), ceftazidime (CAZ: 30 μg), imipenem (IPM: 10 μg), Cefixime (CXM) (30μg), Cefotaxime (CTX) (30μg), Penicillin (PEN) (10 IU), Cloxacillin (CXC) (5μg), ertapenem (ETP: 10 μg), meropenem (MEM: 10 μg), tetracycline (TET: 30 μg), trimethoprim-sulfamethoxazole (SXT: 30μg), gentamicin (GEN: 10μg), nalidixic acid (NAL: 30 μg), ciprofloxacin (CIP: 5 μg), chloramphenicol (CHL: 30 μg), aztreonam (AZT: 15 μg), piperacillin/tazobactam (TZP: 100/10 μg), fosfomycin (FOF: 200 μg), and colistin (CST: 10 μg) were examined using Mueller-Hinton agar antibiotic diffusion technique (Kirby-Bauer NCCLS modified disc diffusion technique). CTX, AMC and CAZ antibiotic discs for double-disk synergy test of ESBLs were also performed. The isolated bacteria were plated on a dry Mueller Hinton agar plate with appropriate antimicrobial-impregnated disks and cultured overnight at 35^o^C. The antibiotic inhibition zones were measured from the centre to the distinct edges of antibiotic inhibition zones using a ruler with *E. coli* ATCC 25922 strains as a control. The AST discs were obtained from Becton Dickinson (BD, Sparks, MD, USA).”

10. Under same subsection, authors have to indicate the source of AST discs used to obtain the susceptibility pattern described in tis study.

Response: We have included the source of AST discs to “he AST discs was obtained from Becton Dickinson (BD, Sparks, MD, USA).”

11. Under Antibiotic Susceptibility Test (AST), reference 33 is misplaced. Place it after … (Kirby-Bauer NCCLS modified disc diffusion technique). I don’t even understand what that ref means, the list of antibiotics indicated were what you tested your isolates against in your lab so the reference? Reading Anning et al, Antibiotic susceptibility pattern of Enterobacteriaceae isolated from raw meat and Ghanaian coin currencies at Cape Coast metropolis, Ghana: the public health implication only indicated the AST profile for some organisms. Justify the reference or delete it from the ms.

Response: Reference 33 has been deleted

12. Sources of reagents reported indicated in this study. it was interesting to note that all the reagents used to obtain the isolates analyzed in this study was obtained from the same source, was it designed or coincidental?

Response: This was not design but rather coincidental as the data was taken from already analyzed samples from OPD.

13. Please provide ethical approval body and reference number for the study

Response: We have included the ethical approval information “**Ethical approval** from the Department of Biomedical Sciences Research Board, University of Cape Coast (DBRB/17/0432) was obtained before the study and permission from the Medical Administrative Committee of University of Cape Coast Hospital. All data obtained from Laboratory records were anonymous.”

Results
1. Demographics: Authors should include clinical sources of the patients whose samples were analyzed, namely, OPD, in-patient, ANC etc and discussion the implications of this in relation to public health

Response: All data is from outpatient department (OPD) as shown in the modified title “**Antibiotic Resistance among pathogenic bacterial isolates from patients attending the Outpatient Department of University of Cape Coast hospital, Ghana: A retrospective study between 2013-2015**”

2. Table 1: I do not get it why you analyzed bacterial pathogens from other sample sources aside urine, HVS and urethral. Based on the focus of your data, there is no need to include pathogens from other samples. Please revise the manuscript

Response: We have revised the manuscript to include all pathogens collected at the OPD

3. The figures used were good but not very clear. Kindly present clear images in your revised manuscript

Response: We have provided the PDF format of the result to make a bit clearer

4. Figure 3D, please define the abbreviations

Response: We have included a defined abbreviation as “Bactrim (BA), tetracycline (TE), ampicillin-sulbactam (AS), clindamycin (CL), ofloxacin (OF), lincomycin (LZ) and chloramphenicol (CH)”

5. It will be of interest to include the year-by-year gender and age incidence trends of the isolates and drug resistant isolates

Response: We have included it in the supplementary information “supplementary information table 1”

6. Authors should provide the ZoIs of the various pathogens regarding each antibiotic as a supplementary material

Response: We have included it in the supplementary information “supplementary information table 2”

Discussion
1. TEM-, SHV-, CTX-M-, and OXA-type are resistance genes, please revise to capture that

Response: we have revised it to “The antibiotic-resistant uropathogens employ several resistance mechanisms including the expressions of β-lactamases, TEM-, SHV-, CTX-M-, and OXA-type resistance genes responsible for extended-spectrum β-lactam resistance (ESBLs) [41, 42]”

2. The results section as well as the discussion section was silent on the gender trend of the DR isolates. Please include them appropriately

Response: We have included the statement “The trends of pathogens isolation were high among females compared to males and the median age between subjects ranging between 20 and 35 years (supplementary information table 1)” to result section.

3. Authors have to understand that various AST disks may give different ZoIs to the same pathogen, hence phenotypic assessment of DR is not conclusive, therefore inclusion of suggestion of molecular surveillance for DR gene markers in pathogens with high ZoIs is essential. Authors can also review some of the genetic markers associated with bacterial DR. Additionally, authors have to discuss some of the reported prevalence of bacterial DR globally, continentally and in some other parts of Ghana.

Response: We have included the “The isolated resistant pathogens exhibited different antibiotic resistance patterns (Supplementary information table 2)” to the result section under the network of interactions of pathogenic bacteria isolates and antibiotic resistance

Reviewer #2: The manuscript entitled “Antibiotic Resistance among Uropathogenic Escherichia coli (UPEC) isolated from patients attending the University of Cape Coast hospital: Retrospective study between 2013-2015” submitted by Asare KK and co-authors describes a retrospective study on microbial pathogens and antibiotic resistance of bacteria isolated from patients attending a university hospital in Ghana during the years 2013-2015. The authors analyzed the data available in the hospital’s registry including demographic (age, gender), biological (biological samples) and microbiological data (pathogen isolated and antibiotic resistance). Overall, the manuscript suffers from several drawbacks that make it unsuitable for publication as it is. Below are specific comments on the content and the quality of the manuscript.

1. The title of the manuscript does not reflect its content. The title mentions a focus on UPEC, while in the results section the authors present an analysis of all the bacterial and Candida infections found in the different biological samples during the study period. I suggest that the authors (i) keep the title and conduct an analysis clearly focused on UPEC isolates found during the study period, or (ii) change the title to more broadly include all the bacterial pathogens isolated.

Response: we have revised the title to “**Antibiotic Resistance among pathogenic bacterial isolates from patients attending the Outpatient Department of University of Cape Coast hospital, Ghana: A retrospective study between 2013-2015**”

2. The English level of the manuscript is poor with too many grammar and syntax errors. The manuscript should be combed through by a writer fluent in English to bring it to a standard suitable for a scientific journal.

Response: We have revised the manuscript to correct the grammatical errors

3. The analysis of bacterial pathogens isolated during the study period is poorly structured with all the data lumped together and very difficult to follow. Analyzing the data in males vs in females, then by age groups is a sound approach. However, these different analyses should be clearly separated in order to show the discoveries. I suggest the authors to divide this analysis to different subsections, each dealing which an aspect, presenting the particular findings and ending with a conclusion.

Response: We have included the trends in pathogenic isolates among males and females in the supplementary information table 1 and antibiotic zones of infection for various antibiotics by the isolated pathogens in the supplementary information table 2

The authors analyzed antimicrobial resistance (AMR) by conducting a network of interactions between pathogenic bacteria and antibiotic resistance. There are several concerns regarding this analysis.

4. Firstly, the methodology of this analysis is not detailed at all in the material and methods section.

Response: we have included in the methodology section a subsection that reads,

“**Diagrammatic presentation of relationships between pathogenic bacterial isolates and antibiotic resistance**

The network tool (Cytoscape) reduces the underlying complexity in pathogens and antibiotic resistance association through diagrammatic representation for concepts and relationships. The analysis assessed the characteristics of the isolated pathogens and antibiotic resistance patterns through metrics measurements such as degree, clustering coefficient, shortest paths, centrality, density. The network analysis provides visualizing of the direct association between pathogens and their antibiotic resistance status. It also estimates pathogens and their trends of antibiotic resistance.”

5. Secondly, the goals of this analysis is not clearly mentioned by the authors anywhere in the manuscript.

Response: We have included “The isolated pathogens and their antibiotic-resistant status were visualized using the network of interactions to understand the magnitude and the trends of antibiotic resistance among species of pathogenic bacterial isolates.”

6. Thirdly, the authors stated findings without telling neither their meaning nor their significance. For example, in the subsection of the results section entitled “The network of interactions of pathogenic bacteria isolates and antibiotic resistance” the authors states the following: “The result showed a network of nodes connecting to other nodes indicating positive correlations between these pathogens and their sensitivity to various antibiotics (Figure 3 A)” (lines 2-4 of the above-mentioned subsection). What does this means in terms of antibiotic susceptibility of the corresponding bacteria to the antibiotic tested? The same question pertains to the following sentence where the autors wrote: “The individual pathogenic isolates formed a separate cluster of interactions (edges) with the various antibiotics and there was no direct interaction between the individual isolated pathogens. E. coli, Citrobacter spp, Klebsiella spp and the other isolated pathogens formed higher centrality in the network of interaction with antibiotic resistance (Figure 3 B).” Given the importance of AMR in public health, I suggest that the authors present a detailed analysis of susceptibility of bacteria (either UPEC or all pathogens) to relevant antibiotics before describing network of interactions while giving sound conclusions.

Response: We have included the statement “This indicates the difference in the levels of antibiotic resistance among the individual pathogens.” to give further explanation

5. The manuscript would much easier to review if it included line numbers.
Below are additional issue that the authors need to address.

Response: Page numbers have been added as directed.

Materials and methods
In the subsection entitled “Clinical sample collection and identification of pathogens”, the authors wrote the following
• “All the samples were spread onto Blood agar (Sigma-Aldrich) and MacConkey agar (Sigma-Aldrich) and incubated at 37°C overnight.” Were the same media used whatever the biological sample processed? Since there were various biological fluids analyzed, please mention the different procedures applied if any.

Response: We have revised the section to “Convenience sampling was used for the selection of samples. The study samples included high vaginal swabs, blood samples, urethral swabs, urine samples, stool samples, cerebrospinal fluids samples, ear swabs and wound samples collected at the University of Cape Coast hospital. The patients’ demographic characteristics such as age and sex were also recorded from the registrar. Blood agar (Sigma-Aldrich) and MacConkey agar (Sigma-Aldrich) were used for the isolation of the pathogens. The incubation conditions were 37^o^C aerobically overnight. The isolates were identified based on colony appearance, Gram stain, catalase-positive, and oxidase negative isolates were further examined. ”

• “The isolates were identified based on colony appearance, Gram stain, catalase-positive, and oxidase negative isolates were further examined [33].” Details on bacterial identification were not provided. In the subsection that followed, the authors mentioned biochemical methods. Please consistently summarize the methods used to identify bacterial species.

Response: We have revised the section to “The bacteria isolates analyzed in this study were obtained using the standard traditional biochemical tests and selective media such as Endo agar (Sigma-Aldrich), MacConkey broth, Simmons citrate agar (Sigma-Aldrich), catalase (Sigma-Aldrich), coagulase (Sigma-Aldrich), oxidase (Sigma-Aldrich), sugar fermentation [Triple sugar iron (TSI) agar] (Sigma-Aldrich), indole (Sigma-Aldrich), citrate utilization (Sigma-Aldrich), urease production (Sigma-Aldrich), and motility tests were used. The *Neisseria gonorrhoeae* confirmatory test was performed using Identicult-Neisseria (Adams Scientific, USA) test [34]. The test depends on beta-galactosidase, gamma-glutamylaminopeptidase and prolyl-hydroxypropyl aminopeptidase and rapid carbohydrate utilization test (RCUT) of clinical isolates of oxidase-positive Gram-negative diplococci*. E. coli* confirmatory test was performed using Sorbitol-MacConkey (SMAC) agar with *E. coli* O157 antiserum or latex reagents (O157 antibody-coated latex and control latex) based on the manufacturer-recommended procedures.”

There are several inconsistencies in the subsection entitled: “Antibiotic Susceptibility Test (AST)”
• The first sentence is not clear. Start by mentioning the use of disc diffusion, then that 0.5 McFarland suspension are used, etc.

Response: We have revised the section to “Antimicrobial susceptibility was done by Kirby–Bauer disc diffusion method on Mueller–Hinton agar. Briefly, the bacteria turbidity was measured by comparing the normal saline emulsified pure colonies to 0.5 McFarland solution. The susceptibility of bacteria to Ampicillin (Amp) (10μg), Gentamicin (GEN) (10μg), Cotrimoxazole (COT) (25μg), Cefuroxime (CRX) (30μg), Erythromycin (ERY) (5μg) and Amikacin (AMK) (30μg), amoxicillin/clavulanic acid (AMC: 30 μg), cefuroxime (CXM: 30 μg), cefotaxime (CTX: 30 μg), ceftazidime (CAZ: 30 μg), imipenem (IPM: 10 μg), Cefixime (CXM) (30μg), Cefotaxime (CTX) (30μg), Penicillin (PEN) (10 IU), Cloxacillin (CXC) (5μg), ertapenem (ETP: 10 μg), meropenem (MEM: 10 μg), tetracycline (TET: 30 μg), trimethoprim-sulfamethoxazole (SXT: 30μg), gentamicin (GEN: 10μg), nalidixic acid (NAL: 30 μg), ciprofloxacin (CIP: 5 μg), chloramphenicol (CHL: 30 μg), aztreonam (AZT: 15 μg), piperacillin/tazobactam (TZP: 100/10 μg), fosfomycin (FOF: 200 μg), and colistin (CST: 10 μg) were examined using Mueller-Hinton agar antibiotic diffusion technique (Kirby-Bauer NCCLS modified disc diffusion technique). CTX, AMC and CAZ antibiotic discs for double-disk synergy test of ESBLs were also performed. The isolated bacteria were plated on a dry Mueller Hinton agar plate with appropriate antimicrobial-impregnated disks and cultured overnight at 35^o^C. The antibiotic inhibition zones were measured from the centre to the distinct edges of antibiotic inhibition zones with a ruler with *E. coli* ATCC 25922 strains as a control. The AST discs were obtained from Becton Dickinson (BD, Sparks, MD, USA).”

• The authors did not mention the standard they used for AST: EUCAST? CLSI? Other?

Response: We have included “were examined using Mueller-Hinton agar antibiotic diffusion technique (Kirby-Bauer NCCLS modified disc diffusion technique).”

• The authors mentioned Cefuroxime twice, one abbreviated as CRX (third line) and the other as CXM (fourth line).

Response: we have deleted “Cefuroxime (CXM)”

• The authors mentioned Cefotaxime twice (fifth and sixth lines)

Response: we have deleted “Cefotaxime (CTX) (30μg)”

• The authors sometimes used a capital letter to begin the names of the drugs and sometimes a small cap letter; additionally, they wrote the abbreviations and the concentrations of the drugs sometimes separately [e.g. (CTX) (30μg)], sometimes together [e.g. (MEM: 10μg)]. Please use a consistent writing method for these molecules.

Response: We have formalized to “(CTX) (30μg)”

• At the end of this subsection, the authors mentioned the use of E. coli ATCC 259122 as a QC strain for AST. Since several antibiotics were tested, what are the other QC strains used for drugs not relevant to E. coli ATCC 25922?

Response: The antibiotic inhibition zones were measured from the centre to the distinct edge of antibiotic inhibition zones with a ruler and using *E. coli* ATCC 25922 strains. For other isolates, resistant isolates from the laboratory were used as a control. **Note:** the genetic background of the laboratory isolates is known.

Reviewer #3: Dear Authors, please make sure that you have got line numbers in your manuscript next time, therefore reviewers can point the changes directly by saying the line number.

Abstract.
Please seperate "spectrum and beta-lactamase"

Response: we revised it to “extended spectrum β-lactamase (ESBL)”

What kind of standard biochemical test are done and why?

Response: We have revised the statement to “Standard biochemical tests including catalase, coagulase, oxidase, sugar fermentation [Triple sugar iron (TSI) agar], indole, citrate utilization, and urease production were performed for all isolates for bacteria species identification”

please mention "patient"
please write full name of E. coli first time and then shorthen it.

Response: We have revised it to read “*Escherichia coli* (*E. coli*)”

spp is not italic.

Response: we have revised spp to “*spp*”

results section has to be written again, because authors mentioned the antimicrobial resistance pattern testing, but there is no results saying about it.

Response: we have included the statement “The isolated resistant pathogens exhibited different antibiotic resistance patterns”

Introduction:
"species" not italic.

Response: we have italicizes all species names

Please seperate a new paragraph from the sentence "About 20%...."

Response: The new paragraph has been separated from the sentence.

Please check all "comma" necessary places in the mansucript for example before "but" "which" etc.

Response: Manuscript has been checked for possible grammatical and punctuation errors."

Please re-write the last sentence of the "Clinical sample collection and identification of pathogens"

Response: We have revised to “Convenience sampling was used for the selection of samples. The study samples included high vaginal swabs, blood samples, urethral swabs, urine samples, stool samples, cerebrospinal fluids samples, ear swabs and wound samples collected at the University of Cape Coast hospital. The patients’ demographic characteristics such as age and sex were also recorded from the registrar. Blood agar (Sigma-Aldrich) and MacConkey agar (Sigma-Aldrich) were used for the isolation of the pathogens. The incubation conditions were 37^o^C aerobically overnight. The isolates were identified based on colony appearance, Gram stain, catalase-positive, and oxidase negative isolates were further examined.

The bacteria isolates analyzed in this study were obtained using the standard traditional biochemical tests and selective media such as Endo agar (Sigma-Aldrich), MacConkey broth, Simmons citrate agar (Sigma-Aldrich), catalase (Sigma-Aldrich), coagulase (Sigma-Aldrich), oxidase (Sigma-Aldrich), sugar fermentation [Triple sugar iron (TSI) agar] (Sigma-Aldrich), indole (Sigma-Aldrich), citrate utilization (Sigma-Aldrich), urease production (Sigma-Aldrich), and motility tests were used. The *Neisseria gonorrhoeae* confirmatory test was performed using Identicult-Neisseria (Adams Scientific, USA) test [34]. The test depends on beta-galactosidase, gamma-glutamylaminopeptidase and prolyl-hydroxypropyl aminopeptidase and rapid carbohydrate utilization test (RCUT) of clinical isolates of oxidase-positive Gram-negative diplococci*. E. coli* confirmatory test was performed using Sorbitol-MacConkey (SMAC) agar with *E. coli* O157 antiserum or latex reagents (O157 antibody-coated latex and control latex) based on the manufacturer-recommended procedures.”

"Staphylococcus epidermidis (50.94%), Neisseria gonorrhoeae (15.09%), Escherichia
coli (9.43%) and Staphylococcus saprophyticus" please shorthened the name of these isolates.

Response: We have revised Staphylococcus epidermidis (50.94%), Neisseria gonorrhoeae (15.09%), Escherichia coli (9.43%) and Staphylococcus saprophyticus to “*S. epidermidis* (50.94%), *N. gonorrhoeae* (15.09%), *E. coli* (9.43%) and *S. saprophyticus* (7.55%)”

"60.0% vs 0.0% and 40.0%" please delete the comma and 0 if the number is integer.

Response: we have revised 60.0% vs 0.0% and 40.0% to “60% vs 0% and 40% vs 50%”

In results section, there is no data regarding the antimicrobial susseptibility test, please include the results.

Response: We have added the antimicrobial susseptibility test results to the supplementary information table 2 “The isolated resistant pathogens exhibited different antibiotic resistance patterns (Supplementary information table 2).”

Discussion:
ESBL is already abreviated.

Response: We have abbreviated all the ESBL

Most of the discussion section seems like the review paper, rather than discussing the results of this study.

Response: We have revised the discussion
