# Supplementary material for: Antibiotic-resistant pathogenic bacterial isolates from patients attending the outpatient department of university of Cape Coast hospital, Ghana: A retrospective study between 2013–2015
Source: PLOS Glob Public Health. 2022 May 16;2(5):e0000417. doi: 10.1371/journal.pgph.0000417 (PMC10021532; doi:10.1371/journal.pgph.0000417)
Supplement: Peer review history

# Peer review: Decision Letter 0

**DOI:** 10.1371/journal.pgph.0000417.r001

**Authors:** Kwabena Obeng Duedu, Julia Robinson

10 Nov 2021

PGPH-D-21-00765

Antibiotic Resistance among Uropathogenic Escherichia coli (UPEC) isolated from patients attending the University of Cape Coast hospital: Retrospective study between 2013-2015

PLOS Global Public Health

Dear Dr. Asare,

Thank you for submitting your manuscript to PLOS Global Public Health. After careful consideration, we feel that it has merit but does not fully meet PLOS Global Public Health’s publication criteria as it currently stands. Therefore, we invite you to submit a revised version of the manuscript that addresses the points raised during the review process.

Please submit your revised manuscript by Dec 25 2021 11:59PM. If you will need more time than this to complete your revisions, please reply to this message or contact the journal office at globalpubhealth@plos.org. When you're ready to submit your revision, log on to https://www.editorialmanager.com/pgph/ and select the 'Submissions Needing Revision' folder to locate your manuscript file.

Please include the following items when submitting your revised manuscript:

- A rebuttal letter that responds to each point raised by the editor and reviewer(s). You should upload this letter as a separate file labeled 'Response to Reviewers'.
- A marked-up copy of your manuscript that highlights changes made to the original version. You should upload this as a separate file labeled 'Revised Manuscript with Track Changes'.
- An unmarked version of your revised paper without tracked changes. You should upload this as a separate file labeled 'Manuscript'.

Guidelines for resubmitting your figure files are available below the reviewer comments at the end of this letter.

We look forward to receiving your revised manuscript.

Kind regards,

Kwabena Obeng Duedu, PhD

Academic Editor

PLOS Global Public Health

Journal Requirements:

1. In your ethics statement in the Methods section and in the online submission form, please provide additional information about the data used in your retrospective study. Specifically, please ensure that you have discussed whether all data from the Microbiology Laboratory register records were fully anonymized before you accessed them.

Additional Editor Comments (if provided):

Revise the discussion and ensure results are adequately discussed.

[Note: HTML markup is below. Please do not edit.]

Reviewers' comments:

Reviewer's Responses to Questions

**Comments to the Author**

1. Does this manuscript meet PLOS Global Public Health’s publication criteria? Is the manuscript technically sound, and do the data support the conclusions? The manuscript must describe methodologically and ethically rigorous research with conclusions that are appropriately drawn based on the data presented.

Reviewer #1: Yes

Reviewer #2: No

Reviewer #3: Yes

**********

2. Has the statistical analysis been performed appropriately and rigorously?

Reviewer #1: Yes

Reviewer #2: No

Reviewer #3: Yes

**********

3. Have the authors made all data underlying the findings in their manuscript fully available (please refer to the Data Availability Statement at the start of the manuscript PDF file)?

The PLOS Data policy requires authors to make all data underlying the findings described in their manuscript fully available without restriction, with rare exception. The data should be provided as part of the manuscript or its supporting information, or deposited to a public repository. For example, in addition to summary statistics, the data points behind means, medians and variance measures should be available. If there are restrictions on publicly sharing data—e.g. participant privacy or use of data from a third party—those must be specified.

Reviewer #1: No

Reviewer #2: Yes

Reviewer #3: Yes

**********

4. Is the manuscript presented in an intelligible fashion and written in standard English?

PLOS Global Public Health does not copyedit accepted manuscripts, so the language in submitted articles must be clear, correct, and unambiguous. Any typographical or grammatical errors should be corrected at revision, so please note any specific errors here.

Reviewer #1: Yes

Reviewer #2: No

Reviewer #3: Yes

**********

5. Review Comments to the Author

Please use the space provided to explain your answers to the questions above. You may also include additional comments for the author, including concerns about dual publication, research ethics, or publication ethics. (Please upload your review as an attachment if it exceeds 20,000 characters)

Reviewer #1: This study is very essential as it reports the frequencies and prevalence of bacterial DR isolates in urogenital specimens in the Central region of Ghana, particularly, in hospital attendees in the UCC Hospital. The use of cytoscape in presentations of data was interesting. However, the figures were not clear (especially Fig 2B, 3A and B) and it will be better if clear single figures are presented for all the images.

Abstract

1. Introduction: separate spectrum from β-lactamase

2. Results: The E. coli prevalence among the age categories were 2/21 (9.52%), 22/114 (19.30%) and 2/15 (13.33%) among ≤20 years, 21-40 years and 41-60 years respectively, the numerators don’t add up to the 32 infections. It looks as if you presented for only the females as the numerator add up to 26 and the denominator adds up to 150. Please recheck and revise

Introduction

1. … their pathogenic threshold of 105 CFU/mL. Indicate the full form of abbreviation on initial use

2. UTIs are the third most common bacterial disease, indicate the first two most common bacterial diseases. This will enrich the background of the study

3. The mechanism of development of bacterial drug resistance was poor explained. Please explain into details mechanisms by which bacteria may become insensitive to antibiotics. The molecular basis was provided but there are human behaviors that encourage bacterial drug resistance.

4. Additionally, review global, continental and local trend of DR and some economic importance of DR regarding bacterial infections

5. The trend and risk factors associated with antibiotic resistance pathogens associated with UTIs require constant, change resistance to resistant

Methods

1. Study clinical sample collection …, authors should indicate the sampling strategy employed in this study whether random or convenient

2. Under the objective of your study was to look at E. coli infections in urosamples. It is not clear why under Clinical sample collection and identification of pathogens, you indicated that pathogens isolated in blood samples, stool samples, cerebrospinal fluids samples, ear swabs and wound samples were also analyzed. Under the same subheading, you indicated that ‘All the samples were spread onto Blood agar (Sigma-Aldrich) and MacConkey agar (Sigma-Aldrich) and incubated at 37°C overnight’. My understanding was that you reviewed laboratory records so its not proper to indicated how the C/S was done in this way. you will have to review that statement to reflect that blood agar (Sigma-Aldrich) and MacConkey agar (Sigma-Aldrich) was used to isolate the pathogen analyzed in this study. They incubation conditions were 37°C aerobically overnight.

3. Under Biochemical Identification, avoid repeating the source of the reagents (Sigma-Aldrich). Revision is required in that regard

4. Change (Triple sugar iron (TSI) agar) to [Triple sugar iron (TSI) agar]

5. MacConkey broth (Sigma-Aldrich), and Simmons citrate agar (Sigma-Aldrich) … no need to insert and between these 2 media while others were to follow

6. As was dine for the other, indicate the source of these reagents; catalase, oxidase, sugar fermentation (Triple sugar iron (TSI) agar), indole, citrate utilization, urease

7. Under Biochemical Identification, revise the section to reflect that, that was the technique used to obtain the isolates used in this study. You may be working in the hospital lab and may be familiar with the protocols for bacteria isolation and identification, you don’t have to indicate as if you purposively isolated these pathogens, remember, this is a retrospective study (analysis of already isolated and identified pathogens)

8. Candida species detection: you can revise this subsection as … Candida spp analyzed in this study was obtained by cultivating suspected specimens on Sabouraud glucose agar (SGA) and subsequently, the isolates were confirmed morphologically using assimilation/fermentation methods or cultured Corn meal-Tween 80 agar (CTA)

9. Under Antibiotic Susceptibility Test (AST), previous comments apply to this section as well

10. Under same subsection, authors have to indicate the source of AST discs used to obtain the susceptibility pattern described in tis study.

11. Under Antibiotic Susceptibility Test (AST), reference 33 is misplaced. Place it after … (Kirby-Bauer NCCLS modified disc diffusion technique). I don’t even understand what that ref means, the list of antibiotics indicated were what you tested your isolates against in your lab so the reference? Reading Anning et al, Antibiotic susceptibility pattern of Enterobacteriaceae isolated from raw meat and Ghanaian coin currencies at Cape Coast metropolis, Ghana: the public health implication only indicated the AST profile for some organisms. Justify the reference or delete it from the ms.

12. Sources of reagents reported indicated in this study. it was interesting to note that all the reagents used to obtain the isolates analyzed in this study was obtained from the same source, was it designed or coincidental?

13. Please provide ethical approval body and reference number for the study

Results

1. Demographics: Authors should include clinical sources of the patients whose samples were analyzed, namely, OPD, in-patient, ANC etc and discussion the implications of this in relation to public health

2. Table 1: I do not get it why you analyzed bacterial pathogens from other sample sources aside urine, HVS and urethral. Based on the focus of your data, there is no need to include pathogens from other samples. Please revise the manuscript

3. The figures used were good but not very clear. Kindly present clear images in your revised manuscript

4. Figure 3D, please define the abbreviations

5. It will be of interest to include the year-by-year gender and age incidence trends of the isolates and drug resistant isolates

6. Authors should provide the ZoIs of the various pathogens regarding each antibiotic as a supplementary material

Discussion

1. TEM-, SHV-, CTX-M-, and OXA-type are resistance genes, please revise to capture that

2. The results section as well as the discussion section was silent on the gender trend of the DR isolates. Please include them appropriately

3. Authors have to understand that various AST disks may give different ZoIs to the same pathogen, hence phenotypic assessment of DR is not conclusive, therefore inclusion of suggestion of molecular surveillance for DR gene markers in pathogens with high ZoIs is essential. Authors can also review some of the genetic markers associated with bacterial DR. Additionally, authors have to discuss some of the reported prevalence of bacterial DR globally, continentally and in some other parts of Ghana.

Reviewer #2: The manuscript entitled “Antibiotic Resistance among Uropathogenic Escherichia coli (UPEC) isolated from patients attending the University of Cape Coast hospital: Retrospective study between 2013-2015” submitted by Asare KK and co-authors describes a retrospective study on microbial pathogens and antibiotic resistance of bacteria isolated from patients attending a university hospital in Ghana during the years 2013-2015. The authors analyzed the data available in the hospital’s registry including demographic (age, gender), biological (biological samples) and microbiological data (pathogen isolated and antibiotic resistance). Overall, the manuscript suffers from several drawbacks that make it unsuitable for publication as it is. Below are specific comments on the content and the quality of the manuscript.

1. The title of the manuscript does not reflect its content. The title mentions a focus on UPEC, while in the results section the authors present an analysis of all the bacterial and Candida infections found in the different biological samples during the study period. I suggest that the authors (i) keep the title and conduct an analysis clearly focused on UPEC isolates found during the study period, or (ii) change the title to more broadly include all the bacterial pathogens isolated.

2. The English level of the manuscript is poor with too many grammar and syntax errors. The manuscript should be combed through by a writer fluent in English to bring it to a standard suitable for a scientific journal.

3. The analysis of bacterial pathogens isolated during the study period is poorly structured with all the data lumped together and very difficult to follow. Analyzing the data in males vs in females, then by age groups is a sound approach. However, these different analyses should be clearly separated in order to show the discoveries. I suggest the authors to divide this analysis to different subsections, each dealing which an aspect, presenting the particular findings and ending with a conclusion.

4. The authors analyzed antimicrobial resistance (AMR) by conducting a network of interactions between pathogenic bacteria and antibiotic resistance. There are several concerns regarding this analysis. Firstly, the methodology of this analysis is not detailed at all in the material and methods section. Secondly, the goals of this analysis is not clearly mentioned by the authors anywhere in the manuscript. Thirdly, the authors stated findings without telling neither their meaning nor their significance. For example, in the subsection of the results section entitled “The network of interactions of pathogenic bacteria isolates and antibiotic resistance” the authors states the following: “The result showed a network of nodes connecting to other nodes indicating positive correlations between these pathogens and their sensitivity to various antibiotics (Figure 3 A)” (lines 2-4 of the above-mentioned subsection). What does this means in terms of antibiotic susceptibility of the corresponding bacteria to the antibiotic tested? The same question pertains to the following sentence where the autors wrote: “The individual pathogenic isolates formed a separate cluster of interactions (edges) with the various antibiotics and there was no direct interaction between the individual isolated pathogens. E. coli, Citrobacter spp, Klebsiella spp and the other isolated pathogens formed higher centrality in the network of interaction with antibiotic resistance (Figure 3 B).” Given the importance of AMR in public health, I suggest that the authors present a detailed analysis of susceptibility of bacteria (either UPEC or all pathogens) to relevant antibiotics before describing network of interactions while giving sound conclusions.

5. The manuscript would much easier to review if it included line numbers.

Below are additional issue that the authors need to address.

Materials and methods

In the subsection entitled “Clinical sample collection and identification of pathogens”, the authors wrote the following

• “All the samples were spread onto Blood agar (Sigma-Aldrich) and MacConkey agar (Sigma-Aldrich) and incubated at 37°C overnight.” Were the same media used whatever the biological sample processed? Since there were various biological fluids analyzed, please mention the different procedures applied if any.

• “The isolates were identified based on colony appearance, Gram stain, catalase-positive, and oxidase negative isolates were further examined [33].” Details on bacterial identification were not provided. In the subsection that followed, the authors mentioned biochemical methods. Please consistently summarize the methods used to identify bacterial species.

There are several inconsistencies in the subsection entitled: “Antibiotic Susceptibility Test (AST)”

• The first sentence is not clear. Start by mentioning the use of disc diffusion, then that 0.5 McFarland suspension are used, etc.

• The authors did not mention the standard they used for AST: EUCAST? CLSI? Other?

• The authors mentioned Cefuroxime twice, one abbreviated as CRX (third line) and the other as CXM (fourth line).

• The authors mentioned Cefotaxime twice (fifth and sixth lines)

• The authors sometimes used a capital letter to begin the names of the drugs and sometimes a small cap letter; additionally, they wrote the abbreviations and the concentrations of the drugs sometimes separately [e.g. (CTX) (30�g)], sometimes together [e.g. (MEM: 10�g)]. Please use a consistent writing method for these molecules.

• At the end of this subsection, the authors mentioned the use of E. coli ATCC 259122 as a QC strain for AST. Since several antibiotics were tested, what are the other QC strains used for drugs not relevant to E. coli ATCC 25922?

Reviewer #3: Dear Authors, please make sure that you have got line numbers in your manuscript next time, therefore reviewers can point the changes directly by saying the line number.

Abstract.

Please seperate "spectrum and beta-lactamase"

What kind of standard biochemical test are done and why?

please mention "patient"

please write full name of E. coli first time and then shorthen it.

spp is not italic.

results section has to be written again, because authors mentioned the antimicrobial resistance pattern testing, but there is no results saying about it.

Introduction:

"species" not italic.

Please seperate a new paragraph from the sentence "About 20%...."

Please check all "comma" necessary places in the mansucript for example before "but" "which" etc.

Please re-write the last sentence of the "Clinical sample collection and identification of pathogens"

"Staphylococcus epidermidis (50.94%), Neisseria gonorrhoeae (15.09%), Escherichia

coli (9.43%) and Staphylococcus saprophyticus" please shorthened the name of these isolates.

"60.0% vs 0.0% and 40.0%" please delete the comma and 0 if the number is integer.

In results section, there is no data regarding the antimicrobial susseptibility test, please include the results.

Discussion:

ESBL is already abreviated.

Most of the discussion section seems like the review paper, rather than discussing the results of this study.

**********

6. PLOS authors have the option to publish the peer review history of their article (what does this mean?). If published, this will include your full peer review and any attached files.

**Do you want your identity to be public for this peer review?** If you choose “no”, your identity will remain anonymous but your review may still be made public.

For information about this choice, including consent withdrawal, please see our Privacy Policy.

Reviewer #1: **Yes: **Dr Enoch Aninagyei

Reviewer #2: **Yes: **Yakhya Dieye

Reviewer #3: No

**********

[NOTE: If reviewer comments were submitted as an attachment file, they will be attached to this email and accessible via the submission site. Please log into your account, locate the manuscript record, and check for the action link "View Attachments". If this link does not appear, there are no attachment files.]

While revising your submission, please upload your figure files to the Preflight Analysis and Conversion Engine (PACE) digital diagnostic tool, https://pacev2.apexcovantage.com/. PACE helps ensure that figures meet PLOS requirements. To use PACE, you must first register as a user. Registration is free. Then, login and navigate to the UPLOAD tab, where you will find detailed instructions on how to use the tool. If you encounter any issues or have any questions when using PACE, please email PLOS at figures@plos.org. Please note that Supporting Information files do not need this step.

# Author comment: Author response to Decision Letter 0

**DOI:** 10.1371/journal.pgph.0000417.r002

4 Dec 2021

Attachment Submitted filename: 2.12.2021 Response to reviewers_Microbiology.doc Click here for additional data file.

# Peer review: Decision Letter 1

**DOI:** 10.1371/journal.pgph.0000417.r003

**Authors:** Kwabena Obeng Duedu, Julia Robinson

8 Apr 2022

Antibiotic-Resistant pathogenic bacterial isolates from patients attending the Outpatient Department of University of Cape Coast hospital, Ghana: A retrospective study between 2013-2015

PGPH-D-21-00765R1

Dear Dr Asare,

We are pleased to inform you that your manuscript 'Antibiotic-Resistant pathogenic bacterial isolates from patients attending the Outpatient Department of University of Cape Coast hospital, Ghana: A retrospective study between 2013-2015' has been provisionally accepted for publication in PLOS Global Public Health.

Before your manuscript can be formally accepted you will need to complete some formatting changes, which you will receive in a follow up email. A member of our team will be in touch with a set of requests.

Please note that your manuscript will not be scheduled for publication until you have made the required changes, so a swift response is appreciated.

IMPORTANT: The editorial review process is now complete. PLOS will only permit corrections to spelling, formatting or significant scientific errors from this point onwards. Requests for major changes, or any which affect the scientific understanding of your work, will cause delays to the publication date of your manuscript.

If your institution or institutions have a press office, please notify them about your upcoming paper to help maximize its impact. If they'll be preparing press materials, please inform our press team as soon as possible -- no later than 48 hours after receiving the formal acceptance. Your manuscript will remain under strict press embargo until 2 pm Eastern Time on the date of publication. For more information, please contact globalpubhealth@plos.org.

Thank you again for supporting Open Access publishing; we are looking forward to publishing your work in PLOS Global Public Health.

Best regards,

Kwabena Obeng Duedu, PhD

Academic Editor

PLOS Global Public Health

***********************************************************

Reviewer Comments (if any, and for reference):

Reviewer's Responses to Questions

**Comments to the Author**

1. If the authors have adequately addressed your comments raised in a previous round of review and you feel that this manuscript is now acceptable for publication, you may indicate that here to bypass the “Comments to the Author” section, enter your conflict of interest statement in the “Confidential to Editor” section, and submit your "Accept" recommendation.

Reviewer #1: All comments have been addressed

Reviewer #2: (No Response)

Reviewer #3: All comments have been addressed

**********

2. Does this manuscript meet PLOS Global Public Health’s publication criteria? Is the manuscript technically sound, and do the data support the conclusions? The manuscript must describe methodologically and ethically rigorous research with conclusions that are appropriately drawn based on the data presented.

Reviewer #1: Yes

Reviewer #2: No

Reviewer #3: Yes

**********

3. Has the statistical analysis been performed appropriately and rigorously?

Reviewer #1: Yes

Reviewer #2: No

Reviewer #3: Yes

**********

4. Have the authors made all data underlying the findings in their manuscript fully available (please refer to the Data Availability Statement at the start of the manuscript PDF file)?

The PLOS Data policy requires authors to make all data underlying the findings described in their manuscript fully available without restriction, with rare exception. The data should be provided as part of the manuscript or its supporting information, or deposited to a public repository. For example, in addition to summary statistics, the data points behind means, medians and variance measures should be available. If there are restrictions on publicly sharing data—e.g. participant privacy or use of data from a third party—those must be specified.

Reviewer #1: Yes

Reviewer #2: Yes

Reviewer #3: Yes

**********

5. Is the manuscript presented in an intelligible fashion and written in standard English?

PLOS Global Public Health does not copyedit accepted manuscripts, so the language in submitted articles must be clear, correct, and unambiguous. Any typographical or grammatical errors should be corrected at revision, so please note any specific errors here.

Reviewer #1: Yes

Reviewer #2: No

Reviewer #3: Yes

**********

6. Review Comments to the Author

Please use the space provided to explain your answers to the questions above. You may also include additional comments for the author, including concerns about dual publication, research ethics, or publication ethics. (Please upload your review as an attachment if it exceeds 20,000 characters)

Reviewer #1: None

Reviewer #2: In an initial review, I made several comments and requested changes needed for the manuscript to be considered for publication. Most of the questions I raised have not been addressed. One important issue is the poor level of English of the manuscript that is still not addressed. Please see attached file for the content of my previous review.

Reviewer #3: Authors have done all necessary changes. So it can be published.

**********

7. PLOS authors have the option to publish the peer review history of their article (what does this mean?). If published, this will include your full peer review and any attached files.

**Do you want your identity to be public for this peer review?** If you choose “no”, your identity will remain anonymous but your review may still be made public.

For information about this choice, including consent withdrawal, please see our Privacy Policy.

Reviewer #1: **Yes: **Dr Enoch Aninagyei

Reviewer #2: **Yes: **Yakhya Dieye

Reviewer #3: No

**********
